# Development of an Online Isotope Dilution CE/ICP–MS Method for the Quantification of Sulfur in Biological Compounds

**DOI:** 10.1021/acs.analchem.3c03553

**Published:** 2024-01-31

**Authors:** Dariya Tukhmetova, Jan Lisec, Jochen Vogl, Björn Meermann

**Affiliations:** † Federal Institute for Materials Research and Testing (BAM), Division 1.1—Inorganic Trace Analysis, Richard-Willstätter-Str. 11, 12489 Berlin, Germany; ‡ Federal Institute for Materials Research and Testing (BAM), Division 1.7—Organic Trace and Food Analysis, Richard-Willstätter-Str. 11, 12489 Berlin, Germany

## Abstract

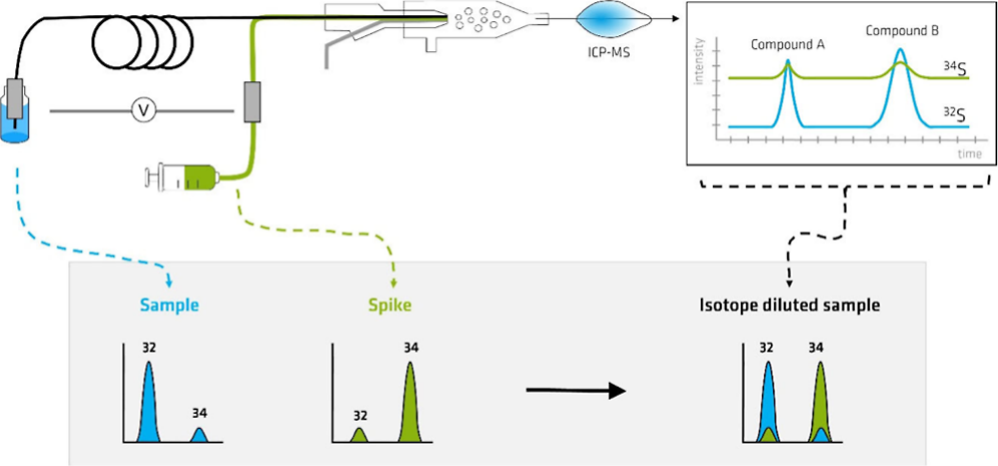

We report an analytical methodology for the quantification of sulfur in biological molecules via a species-unspecific postcolumn isotope dilution (online ID) approach using capillary electrophoresis (CE) coupled online with inductively coupled plasma–mass spectrometry (online ID CE/ICP–MS). The method was optimized using a mixture of standard compounds including sulfate, methionine, cysteine, cystine, and albumin, yielding compound recoveries between 98 and 105%. The quantity of sulfur is further converted to the quantity of the compounds owing to the prior knowledge of the sulfur content in the molecules. The limit of detection and limit of quantification of sulfur in the compounds were 1.3–2.6 and 4.1–8.4 mg L^–1^, respectively, with a correlation coefficient of 0.99 within the concentration range of sulfur of 5–100 mg L^–1^. The capability of the method was extended to quantify albumin in its native matrix (i.e., in serum) using experimentally prepared serum spiked with a pure albumin standard for validation. The relative expanded uncertainty of the method for the quantification of albumin was 6.7% (*k* = 2). Finally, we tested the applicability of the method on real samples by the analysis of albumin in bovine and human sera. For automated data assessment, a software application (IsoCor)—which was developed by us in a previous work—was developed further for handling of online ID data. The method has several improvements compared to previously published setups: (i) reduced adsorption of proteins onto the capillary wall owing to a special capillary-coating procedure, (ii) baseline separation of the compounds in less than 30 min via CE, (iii) quantification of several sulfur species within one run by means of the online setup, (iv) SI traceability of the quantification results through online ID, and (v) facilitated data processing of the transient signals using the IsoCor application. Our method can be used as an accurate approach for quantification of proteins and other biological molecules via sulfur analysis in complex matrices for various fields, such as environmental, biological, and pharmaceutical studies as well as clinical diagnosis.

Sulfur is an essential element in living organisms, where it plays important roles in various biological processes, such as protein synthesis, enzyme activity, and antioxidant defense. However, the biological effects of different sulfur species can vary widely, and imbalances in sulfur speciation have been observed in a range of diseases, including cancer, Alzheimer’s disease, and diabetes.^1−3^ The accurate quantification of sulfur and its species in biological samples requires sensitive and selective analytical techniques. In recent years, separation techniques coupled online with inductively coupled plasma–mass spectrometry (ICP–MS) have emerged as powerful online analytical tools—complementary to molecular spectrometric methods—for speciation analysis of biological compounds.

External calibration^4−9^ and isotope dilution (ID)^10−15^ are common calibration approaches applied for online quantification of sulfur species in complex samples. The ID analysis is advantageous over external calibration because measurement accuracy is largely unaffected by the sample matrix. ID analysis is a highly accurate and precise method for quantification, which involves the addition of a known amount of an isotopically enriched standard, the so-called spike, to the sample, followed by the measurement of the isotope ratios of the spiked and unspiked sample. Depending on the type of spike added to the sample, ID analysis can be performed in two modes: species-specific spiking mode and species-unspecific spiking mode. In the species-specific spiking mode, isotopically labeled forms of the target species are mixed with the sample at the beginning of the analytical procedure. For example, the amyloid β peptide, a biomarker for Alzheimer’s disease, was characterized accurately via ID analysis in the species-specific mode via high-performance liquid chromatography (HPLC)/ICP–MS/MS.^14^ In this study, the authors used ^34^S-labeled yeast hydrolysate to prepare spike standards and quantified sulfur-containing amino acids methionine and cysteine after oxidation and hydrolysis of the peptide. In the species-unspecific spiking mode, the so-called postcolumn spiking, the spike is added after the separation step. Here, the molecular form of the spike does not have to be the same as the target compounds, which allows the usage of more accessible generic spike standards instead of scarce and expensive species-specific spike standards. Moreover, postcolumn species-unspecific spiking can be helpful for nontargeted speciation analysis. Schaumlöffel et al.^15^ characterized and quantified metallothionein isoforms at the species-unspecific spiking mode via capillary electrophoresis (CE)/ICP–MS using a mixture of ^34^S, ^65^Cu, ^68^Zn, and ^116^Cd standards added to a makeup liquid after the separation step. Wang et al.^11^ developed an online size exclusion chromatography ICP–MS method with postcolumn species-unspecific spiking for the absolute quantification of sulfur in bovine serum albumin (BSA), superoxide dismutase, and metallothionein-II proteins. In addition to ^34^S, the spike solution also contained ^65^Cu and ^67^Zn, which allowed quantification of S, Cu, and Zn and estimation of S/Cu and S/Zn ratios in the proteins. Feng et al.^10^ employed high-performance liquid chromatography HPLC/ID–ICP–MS to measure sulfur for the quantification of Alzheimer’s disease biomarker amyloid-beta, which is a candidate for a certified reference material (CRM). The authors found good consistency between HPLC/ID–ICP–MS and primary protein quantification method LC/ID–MS.

Despite successful applications of online ID analysis for the characterization of sulfur-containing molecules, the number of publications in this area remains limited. Most of the currently available studies predominantly rely on analysis of standard samples to report their findings. Consequently, there is a significant need to demonstrate the practicality of online methods for analyzing real-life samples. In our research paper, we present an online method for quantifying proteins within their native matrix (albumin in serum matrix) without requiring any prior sample preparation.

Our methodology encompasses the optimization of the quantification process using a mixture of sulfur-containing inorganic and biological molecules (including sulfate, methionine, cysteine, and albumin). We further validate our method by analyzing an experimentally prepared serum sample that was prepared by spiking a pure albumin standard reference material. Additionally, we extended the application of our method to real serum samples obtained from bovine and human blood. Throughout this account, we thoroughly discuss the challenges encountered during instrumental setup, measurement parameter determination, and data processing. We also present effective solutions that address these challenges, ensuring the reliability and robustness of our method.

## Experimental Section

### Chemicals

Purified water was obtained from a Milli-Q water purification system (Merck Millipore, France). Certified standards of sulfur compounds, namely, ICP standard Certipur grade 1000 mg L^–1^ sulfur as (NH_4_)_2_SO_4_ in water, l-methionine CRM, l-cysteine CRM, and amino acid (AA) mix solution CRM, all in TraceCERT grade from Merck KGaA, Germany, and BSA 7% solution 927 standard reference material (SRM) from the National Institute of Standards & Technology (NIST SRM 927 BSA), USA, were used for method optimization and validation. Albumin was chosen as a model protein due to its relatively high sulfur content (1.88%), its known AA sequence for sera of various animals, and, most importantly, its certified total protein content in the pure BSA SRM from NIST which enables method optimization and validation. The NIST SRM 3154 (0.1% H_2_SO_4_) was used for the instrumental mass bias correction. The ^34^S-enriched solution prepared by dissolving elemental sulfur enriched in ^34^S (99.8%) from Trace Sciences International Inc., USA, was used for ID measurement.^16^ Formic acid 98–100% Suprapur from Merck KGaA, Germany, was used to prepare the background electrolyte (BGE) for CE separation. Bromocresol green (BCG) purchased from Alfa Aesar, Germany, was used to determine the albumin content of serum samples. Hexadimethrine bromide ≥95% (Polybrene) and dextran sulfate sodium salt 40,000 g mol^–1^ from Merck KGaA, Germany, were used for the CE capillary coating. 2-Propanol LC–MS grade from CHEMSOLUTE Th. Geyer GmbH & Co. KG, Germany, was added to the CE sheath liquid to decrease the surface tension for better aerosol formation during CE/ICP–MS.

Depletion of albumin from bovine serum was accomplished using depletion columns packed with BSA IgY beads from GenWay BioTech Inc., USA. After albumin depletion, serum proteins were filtered and concentrated using an Amicon Ultra-4 Centrifugal Filter Unit with Ultracel 10 kDa molecular weight cutoff from Merck KGaA, Germany. Bovine and human male sera were obtained from Merck KGaA, Germany. For the online analysis, the serum samples were diluted four times with Milli-Q water. Certificate of analysis from the manufacturer included the albumin content for bovine serum determined via electrophoretic profiling, but the albumin content was not reported for human serum.

### Instrumentation

CE/ICP–MS interfacing was established using a MiraMist CE nebulizer from Burgener Research Inc., Canada, with a sheath liquid. Postcolumn spiking of the ^34^S-enriched isotope standard was accomplished by adding a known amount of ^34^S to the sheath liquid and delivering it at a constant flow rate via a syringe pump (Harvard Apparatus, USA) using a 10 mL Hamilton glass syringe (Hamilton Company, USA). Detailed operating conditions are given in Table 1. The mass flow of the spike was determined gravimetrically by weighing the sheath liquid pumped in certain time intervals.

**Table 1:** Operating Conditions of the CE/ICP–MS Online System

parameter	value
ICP–MS instrument	Element2 sector-field ICP–MS (Thermo Scientific, Germany)
cones	Ni “X” skimmer cone, Ni jet sample cone (Thermo Scientific, Germany)
acquisition	^32^S and ^34^S, with 0.002 s integration time, at medium resolution
	dead time (32 ns) applied automatically with ICP–MS software
gas flow	0.4 L min^–^^1^ sample gas (makeup gas), 1 L min^–^^1^ auxiliary gas
CE instrument	Agilent CE 7100 (Agilent Technologies, Germany)
capillary	50 μm i.d., 90 cm long fused silica with SMIL coating
background electrolyte	0.5 mol L^–^^1^ formic acid, pH ≈ 2
CE injection	hydrodynamically at 100 mbar for 10 s (21–22 nL)
CE run	–30 kV (25–27 μA) with 5 mbar internal pressure at 23°C (change of internal pressure discussed below)
CE postrun	flush with BGE for 180 s, wait 120 s
nebulizer	Burgener MiraMist CE (Burgener Research Inc., Canada)
gas pressure	Ar at 6.5 bar (95 psi)
spray chamber	8 mL Quartz, drainless with makeup gas
sheath liquid	0.01 mol L^–^^1^ HNO_3_ with 5% (v/v) 2-propanol spiked with 0.5 mg kg^–^^1^^34^S in sulfate form
sheath liquid delivery	At 10 μL min^–^^1^ flow with syringe pump

### Procedures

The CE-fused silica capillary was coated with a successive multiple ionic polymer layer (SMIL) coating consisting of two layers of Polybrene separated by a layer of dextran sulfate. Detailed explanation of the coating procedure can be found in Faßbender et al.^17^

The reference sample for the method validation is prepared as follows: two GenWay prepacked columns were used to deplete albumin from bovine serum. The depletion process was repeated four times to obtain approximately 4 mL of albumin-depleted serum proteins. The proteins were concentrated approximately 20-fold using Amicon centrifugal filtration units. The depletion and concentration procedures were performed according to the manufacturer’s protocol. The concentrated proteins were mixed with a known amount of NIST SRM 927 BSA to obtain a mixture of albumin and albumin-depleted serum proteins close to natural bovine serum composition. The prepared sample was denoted as *serum-RM*. Two separate *serum-RM* samples were prepared to ensure the absence of any mistakes or errors during the preparation. According to the manufacturer’s recommendation, the albumin depletion columns can be reused up to 20 times. However, after using 15 times, the depletion efficiency of the columns is reduced from >95% down to >90%;^18^ thus, only two *serum-RM* samples were prepared.

Albumin content analysis with the BCG dyeing method^19^ based on the spectrometric measurement of the absorbance of the BCG–albumin complex at 630 nm wavelength was performed for bovine and human sera. The purpose of the analysis was to compare results from BCG and online ID CE/ICP–MS method for the quantification of albumin. The measurement was performed on a Specord 210 Plus, Analytik Jena GmbH+Co. KG, Germany. Calibration solutions were prepared by diluting NIST SRM 927 BSA at concentrations between 10 and 50 g L^–1^ to quantify albumin in subsequent samples. To avoid complexation of BCG with globulins and overestimation of albumin, the measurement was performed immediately within 30 s after the dye was mixed with serum samples. The calibration curve generated using BSA was used to estimate human serum albumin (HSA), as quantification of HSA using BSA standards was confirmed to be reasonably reliable.^20^

The correction factor for instrumental mass bias was determined by injecting NIST SRM 3154 into CE/ICP–MS via the sheath liquid without the addition of ^34^S, then measuring the intensities of the ^32^S and ^34^S isotopes, and calculating the ^32^S/^34^S ratio via the peak area integration method.^21^ The measured value was compared with the “best estimate for the true” value, which was determined to be ^32^S/^34^S = 22.555 by thermal ionization mass spectrometry (TIMS) previously.^16^ The mass bias measurements were carried out before and after each sample measurement sequence, and then the averaged correction factor from 12 repetitions was used for the data processing.

### Data Processing

The IsoCor application^22^ was extended and used to convert isotope intensities to mass flow from raw text files exported from the ICP–MS instrument (https://bam.de/IsoCor). The calculation included several steps, namely, isotope ratio calculation, instrumental mass bias correction, mass flow calculation, blank correction, and peak integration. The ID equation was adapted from Rottmann and Heumann^23^ and Vogl and Pritzkow,^24^ resulting in a mass flow (MF) electropherogram in ng min^–1^ (see eq 1):
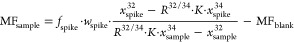
1

The isotope ratio of ^32^S and ^34^S *R*^32/34^ was corrected for instrumental mass bias factor *K* using Russell’s equation^25^ prior to MF calculation. *w*_spike_ and *f*_spike_ are the mass fraction of ^34^S in the sheath liquid (0.5 mg kg^–1^, prepared gravimetrically) and the flow rate of the sheath liquid (10 mg min^–1^, determined gravimetrically), respectively. The isotopic composition of the ^34^S-enriched spike (*x*_spike_^34^ and *x*_spike_^32^) was assigned as 99.8 and 0.2% respectively, according to data previously reported by Pritzkow et al.^16^ Abundance of isotopes in the samples (*x*_sample_^34^ and *x*_sample_^32^) was taken as natural isotopic abundance of organic sulfur of animal origin from the IUPAC Commission on Isotopic Abundances and Atomic Weights (CIAAW).^26^ MF_blank_ was estimated and subtracted during data processing with the IsoCor application. The integration of peaks from the MF electropherogram provided the absolute amount of sulfur in each peak (in ng). Then, the absolute mass was used to calculate sulfur and compound concentrations. The mass concentration of the compound, ρ_compound_, was calculated according to eq 2, where ∑MF_compound_·Δ*t* denotes the integration of the peak, *V*_inj_ is an injected volume, and *M*_compound_ and *M*_sulfur_ are the molecular masses of the compound and sulfur, respectively. The molecular mass of sulfur is taken from CIAAW,^26^ the molecular mass of NIST SRM 927 BSA was obtained from the certificate provided by NIST, the molecular masses of BSA and HSA were obtained from Uniprot.org(27,28) calculated based on a known AA sequence. The injected volume, *V*_inj_ was determined by injecting a sulfate standard solution with a known mass concentration of sulfur and applying 50 mbar of internal pressure to elute the sulfate from the capillary. The peak area was converted to the absolute mass of sulfur, and then *V*_inj_ was found from the known concentration of sulfur in the standard solution (see eq 2).
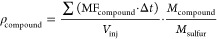
2

Analytical figures of merit [recovery, limit of detection (LOD), limit of quantification (LOQ), and correlation coefficient (*R*^2^)] were evaluated from measurements of sulfate–albumin mixtures with nominal sulfur mass concentrations of 5, 10, 20, 50, and 100 mg L^–1^ and diluted solutions of methionine and cystine in an AA mixture with nominal sulfur mass concentrations of 5, 10, 20, 40, and 80 mg L^–1^. LOD and LOQ are calculated by multiplying the standard error of the calibration curve by factors of 3 and 10, respectively, and then dividing it by the slope. Accuracy was determined by measuring the sulfate–albumin mixture and the AA mixture solutions at the highest concentration from the above-mentioned range.

The complete uncertainty budget of the measurement of albumin concentration in *serum-RM* sample via online ID CE/ICP–MS was calculated for each replicate according to the ISO/GUM guide^29^ using GUM Workbench Pro (Metrodata GmbH, Weil am Rhein, Germany) on eqs 1 and 2. Two types of components of uncertainty contributed to the total budget: type A uncertainty determined from statistical analysis as a standard error of repeated measurements and type B uncertainty found from either manufacturer’s certificate or scientific judgment from already published data. The calculated uncertainty of each measurement, *u*_*i*_, and the standard deviation, *s*, of samples from *n* replicate measurements were combined to calculate the combined standard uncertainty, *u*_c_, as follows^30^ (eq 3)
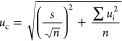
3

A coverage factor of *k* = 2 (95% confidence) was used to calculate the expanded uncertainty, *U* = *k*·*u*_c_.

## Results and Discussion

### Optimization of the CE Separation Method

**Figure 1:**
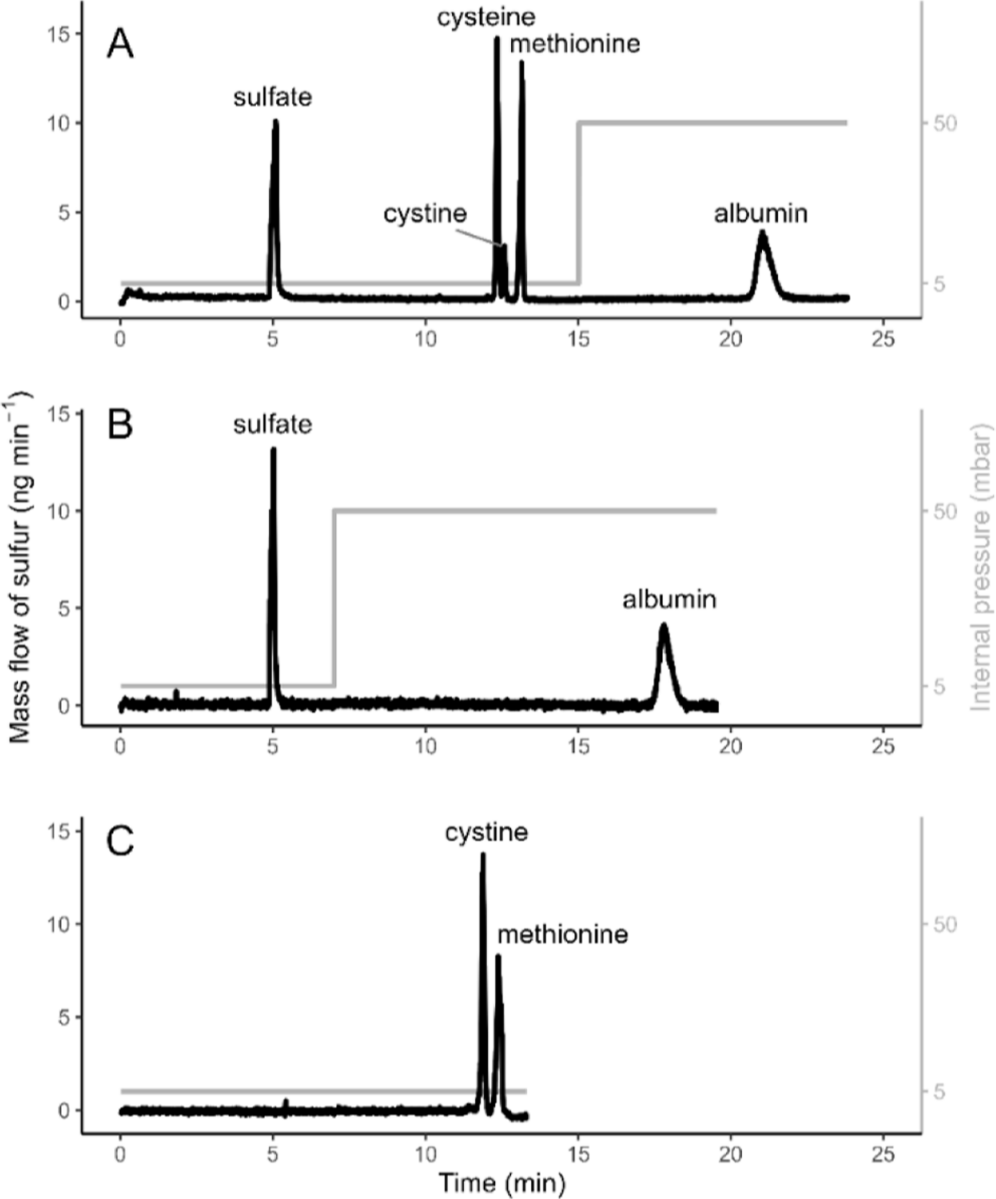
Mass flow electropherograms of the separation of sulfur-containing molecules. (A) Mixture of sulfate, cysteine, methionine, and albumin standards with a nominal mass concentration of sulfur of 100 mg L^–1^; (B) mixture of sulfate and albumin standards with a nominal mass concentration of sulfur of 100 mg L^–1^; and (C) AA standard mixture that contains methionine and cystine with a nominal mass concentration of sulfur of 80 mg L^–1^.

Electrophoretic profiling of serum proteins with CE is a common procedure in clinical chemistry to diagnose immunodeficiency, liver disease, and others.^31,32^ However, several separation parameters of the clinical CE method^33^ are not compatible with ICP–MS and had to be modified as described below: (i) borate or phosphate buffers are not volatile and might clog or precipitate onto the nebulizer or ICP cones, (ii) a 17–20 cm-long capillary is too short to establish a connection between CE and ICP–MS units, and (iii) a 25 μm capillary i.d. is too narrow to inject a sufficient amount of sample into the ICP–MS for optimal sensitivity. The typical length of a capillary used for interfacing CE with ICP–MS is between 70 and 100 cm. However, with long capillaries, the separation efficiency might be compromised due to lowered electric field strength. Furthermore, the migration time is increased, leading to enhanced adsorption of proteins onto the capillary wall. To reduce this adsorption, we opted for SMIL coating of the capillary of 90 cm length with 50 μm i.d. Along with reduced adsorption, SMIL-coated capillaries improve separation efficiency and resolution, as well as increase reproducibility in capillary electrophoresis.^34^ Two volatile organic acids (acetic acid and formic acid) were tested as BGE, and 0.5 mol L^–1^ formic acid was optimal for an efficient separation of sulfur-containing AAs and albumin (Figure 1A). The sulfur species were baseline-separated in less than 25 min. In a related paper from Yeh et al.,^9^ sulfur-containing AAs (l-cysteine, l-cystine, dl-homocysteine, and l-methionine) were separated in less than 8 min by applying a BGE comprising 10 mmol L^–1^ borate buffer at pH 9.8 using a 75 μm i.d. 70 cm-long capillary. In our setup, the acidic pH of BGE and a slightly longer capillary (90 cm) decreased the electroosmotic flow and thus increased the migration time of the AAs. However, SMIL coating of the capillary wall and application of internal pressure (5 mbar) during the separation allowed us to achieve AA separation in less than 13 min. To shorten the migration time of albumin, higher internal pressure (50 mbar) was applied at minute 15 after sulfate and AAs migrated from the capillary (Figure 1A). When the standard solution mixture contained only sulfate and albumin, 50 mbar pressure was applied after minute 7, allowing for faster elution of the albumin peak (Figure 1B).

In Figure 1A, the small peak adjoint to the tail of the cysteine peak is cystine that resulted from thiol-oxidation of cysteine due to its instability.^35^ Initially, all the four sulfur-containing compounds (sulfate, cysteine, methionine, and albumin) were mixed into one standard solution for recovery analysis. However, we observed lowered recovery of AAs and increased recovery of albumin, whereas total recovery of all the four compounds was in between 97.0 and 99.3% (1 s, *n* = 5). This phenomenon can be explained by the high affinity of albumin toward other organic molecules, thus binding with AAs in the mixture solution.^36^ To obtain more accurate recovery results and to prevent interactions between the molecules, albumin was mixed with only sulfate (Figure 1B). A certified solution containing 0.300 mg of g^–1^ cystine and 0.372 mg of g^–1^ methionine together with 15 other AAs was used to determine the recovery of the sulfur-containing AAs (Figure 1C). The recovery and further analytical measurement results are discussed below.

### Quantification of Standard Mixture with Online ID CE/ICP–MS

Two aqueous standard solutions, namely, a sulfate–albumin mixture (Figure 1B) with a sulfur mass concentration of 100 mg L^–1^ in each compound and an AA mixture with a sulfur mass concentration of 80 mg L^–1^ for cystine and for methionine (Figure 1C), were used to evaluate the recovery of the corresponding compounds. The recovery was calculated as the ratio between a prepared concentration and a measured concentration from repeated measurements. All the four compounds were quantitatively recovered, yielding 98.5–105% recovery within the associated uncertainties expressed as 2 times standard deviation (2 s) from six repeated measurements (Table 2). Next to the recovery, further figures of merit of the developed method were determined (*R*^2^, LOD, and LOQ) and are also shown in Table 2. The LOD and LOQ values for sulfur in the compounds differ due to differences in the peak shape and the subsequent peak integration. The estimated sulfur LOD of 2.6 mg L^–1^ S determined for albumin was 160 times higher compared to a previously reported value of 16 μg L^–1^ S from ultraperformance liquid chromatography (UPLC) ID ICP–MS/MS based on the determination of sulfur in the cell-penetrating peptide penetratin.^13^ High discrepancy between these two LOD values can be explained by two factors: (i) the injected volume with CE (21.6 nL) is much lower than that with LC (5 μL); (ii) in our method, a sector field ICP–MS system in medium resolution mode was used—higher mass resolution improves signal-to-noise ratio but also reduces signal intensity. However, when calculating the LOD of absolute amounts of sulfur, the value of the ID CE/ICP–MS method (55.1 pg S) is comparable with the above-mentioned ID LC–ICP–MS/MS method (76.9 pg S).

**Table 2:** Analytical figures of Merit of the Online ID CE/ICP–MS Method

compound	mass fraction of S in the compound (%)	recovery ± 2 s, *n* = 6 (%)	*R*^2^	LOD of S (mg L^–^^1^)	LOD of S, (pg)	LOQ of S (mg L^–^^1^)
sulfate	33.4	100.7 ± 3.0	0.9994	1.3	27.2	4.1
albumin	1.88	99.5 ± 3.2	0.9985	2.6	55.1	8.4
cystine	26.7	105.0 ± 6.6	0.9999	2.4	52.4	8.0
methionine	21.5	98.5 ± 3.4	0.9995	1.5	31.9	4.9

Metrological traceability of measured values to the SI unit was ensured by using an unbroken chain of calibrations. The sulfur mass fraction of the ^34^S spike was determined by reverse ID TIMS using a primary calibrator, NIST SRM 3154, which has been measured with the NIST primary measuring system.^16^ Thus, the sulfur mass fraction in the spike solution is traceable to the SI. After assessing the flow rate of the sheath liquid, that contains the spike, the spike served as a secondary calibrator for online ID CE/ICP–MS measurements, enabling SI-traceable sulfur mass concentrations.

### Validation of the Method with the Reference Material

**Figure 2:**
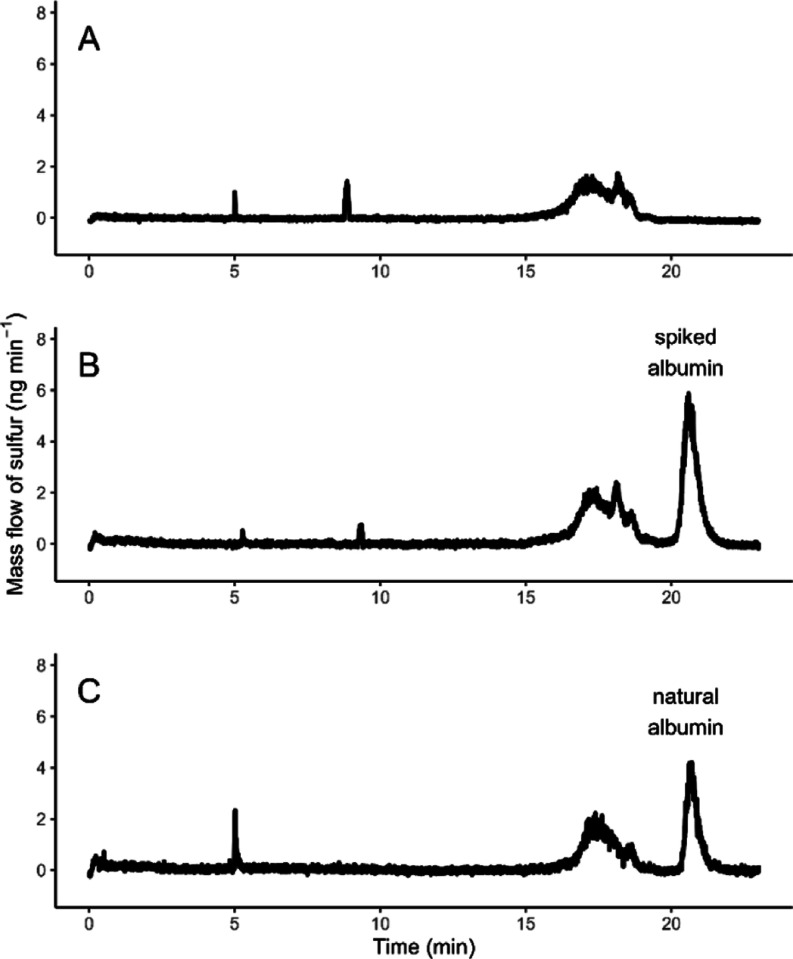
Mass flow electropherogram of sample preparation steps for method validation: (A) serum after albumin depletion (*serum-depleted*); (B) *serum-depleted* spiked with NIST SRM 927 BSA (*serum-RM*); and (C) natural serum (*serum-natural*).

Further investigations concerned the quantification of albumin in a native matrix, i.e., in serum. The *serum-RM* sample with a precise content of albumin (through the spiking of NIST BSA 927 BSA) was used to validate the analytical procedure for quantifying albumin in the experimentally prepared bovine serum sample. According to a quality control study performed by Seam et al.,^18^ the chicken IgY antialbumin microbeads packed into albumin depletion columns from GenWay Biosciences proved to remove >95% albumin from bovine serum. As shown in Figure 2A, after albumin was depleted from the bovine serum matrix, the visual inspection of the MF electropherogram of the *serum-depleted* sample indicated the absence of the albumin. Moreover, the integration of the region where albumin elutes resulted in a value below the LOD. By spiking of NIST SRM 927 BSA into the *serum-depleted* sample, the *serum-RM* sample was prepared. The MF electropherogram of the *serum-RM* sample shows an albumin peak which eluted after other proteins (Figure 2B). Electrophoretic profiling of the *serum-RM* sample is comparable and mimics the composition of the *serum-natural* sample (Figure 2C). Two independent repeated preparations of *serum-RM* were measured with online ID CE/ICP–MS to quantify albumin and compare this value with the spiked concentration of albumin. The results are listed in Table 3. Combined measurement uncertainty *u*_c_ of the albumin mass concentration corresponding to the gravimetric preparation was calculated by taking the uncertainty of NIST SRM 927 BSA, the uncertainty from the albumin depletion efficiency, the uncertainty of albumin recovery, and the uncertainty of weighing into account. The precision of the albumin mass concentration determined via the online ID CE/ICP–MS method is expressed as 2 times standard deviation of the six repeated measurements of each *Serum-RM* sample. Considering the precision of the measured concentration and uncertainty of the prepared concentration of albumin, the values are in agreement, which proves the applicability of our method for a reliable quantification of albumin in serum matrix.

**Table 3:** Mass Concentration of Albumin in the *Serum-RM* Preparations

*Serum-RM* preparation	from gravimetric preparation, ρ ± *u*_c_ (g L^–^^1^)	from online ID CE/ICP–MS measurement, ρ ± 2 s, *n* = 6 (g L^–^^1^)
1	17.6 ± 0.5 (3.0%)	18.1 ± 0.8 (4.4%)
2	11.1 ± 0.3 (3.1%)	11.1 ± 0.8 (7.2%)

### Combined Uncertainty Calculation

**Figure 3:**
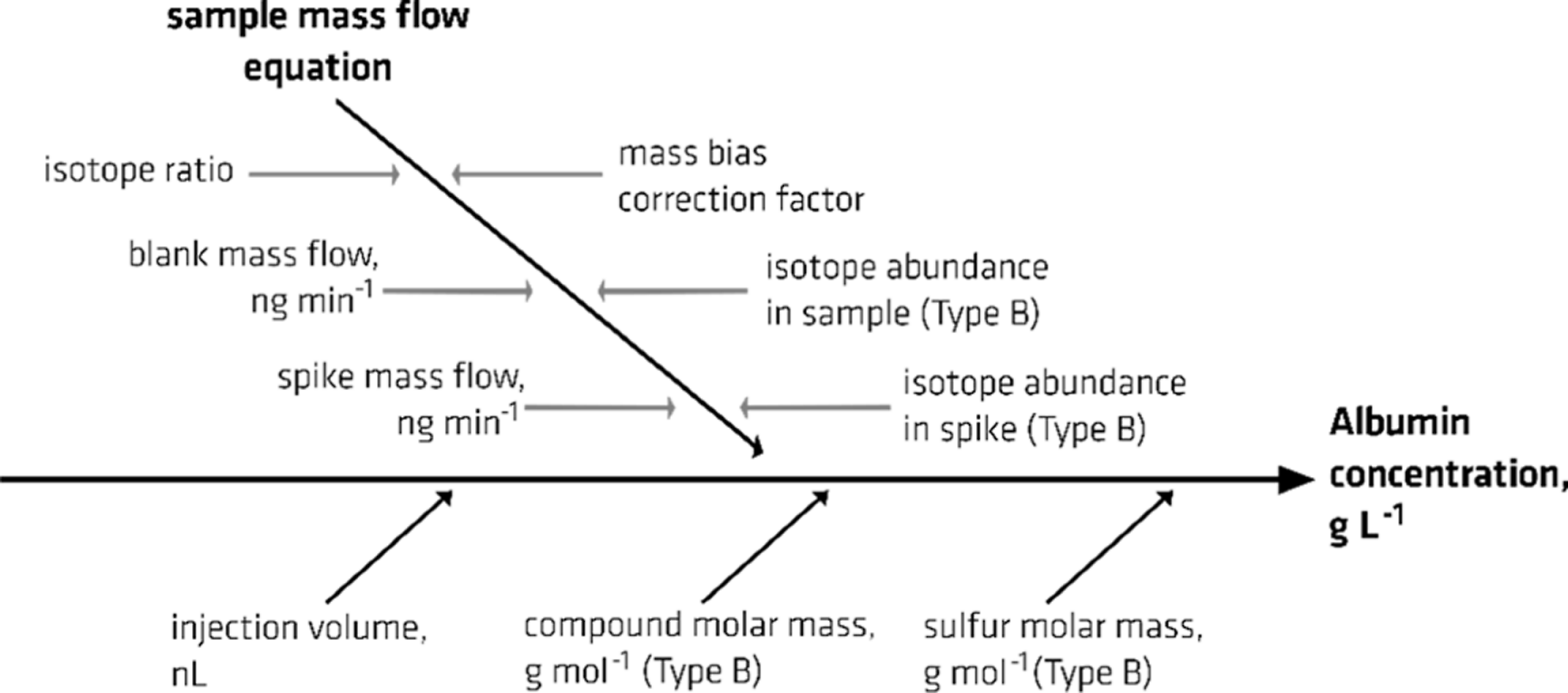
Sources of uncertainty contributing to the total uncertainty of the online ID CE/ICP–MS method.

With the setting up of a complete uncertainty budget for the mass concentration of albumin in the *serum-RM* sample, we identified the main uncertainty contributors. Figure 3 shows all quantities that contribute to the measurement uncertainty. The figure also indicates contributors with Type B uncertainties. The combined uncertainty of a single measurement is 3.1%, which is in-between the relative standard deviation values of two *RM-sample* measurements (Table 3).

As shown in Table 4, the gold standard method for protein quantification—the AA analysis using ID molecular mass spectrometry coupled with liquid chromatography (AAA LC ID–MS)—provides accurate, highly precise (0.8 and 2.0%), and SI-traceable results. However, due to limited availability of sufficiently characterized protein standards in the market, the ID ICP–MS method^30^ is a suitable alternative, with higher uncertainty (3.6%), by using the generic sulfate standard for the characterization and quantification of the protein standards via elemental sulfur analysis. The ID ICP–MS method was based on the analysis of sulfur, which involved the acid digestion of albumin to obtain protein-bound sulfur in the sulfate form after the separation of the nonprotein-bound sulfur by membrane filtration. In this work, although with even higher uncertainty (6.7%), our online ID CE/ICP–MS method enables an online quantification method of albumin in its native matrix (in serum), enabling the direct analysis of sulfur in protein mixtures without the need for sample preparation.

**Table 4:** Comparison of Protein Quantification Methods Applied to NIST SRM BSA

method	target compound	matrix	relative uncertainty *U*, *k* = 2 (%)	reference
online ID CE/ICP–MS	albumin	serum	6.7	this work
ID ICP–MS	albumin	aqueous standard solution	3.6	Lemke et al.^30^
AAA ID LC–MS	albumin	aqueous standard solution	2.0	NIST SRM 927e BSA
AAA ID LC–MS	albumin	aqueous standard solution	0.8	NIST SRM 927f BSA

The top three uncertainty contributors are isotope ratio (69%), mass bias correction factor (13%), and injected volume (11%). The poor precision of the first two parameters can be explained by the influence of several factors: (i) transient signals (in particular from CE) generated with an online system are within a time window of several seconds, thus limiting the number of points for the calculation; (ii) with online systems, the compounds are eluted in the shape of peaks with varying isotope intensities allocating different signal-to-noise ratios to points; and (iii) last, the sample volume injected via CE is significantly lower than the amount of sample measured with conventional systems. The contributing uncertainty of the injected volume (hydrodynamic injection) can be explained by the challenge of optimizing the leveling of the capillary tips when the outlet tip is directed toward ICP–MS. Moreover, the temperature of the capillary is affected by the temperature of the room; thus, a slight change of the temperature leads to the change of the buffer viscosity, consequently resulting in the change of the injected volume. This variation of the injected volume can be minimized using an internal standard.

### Quantification of BSA and HSA in the Native Matrix

**Figure 4:**
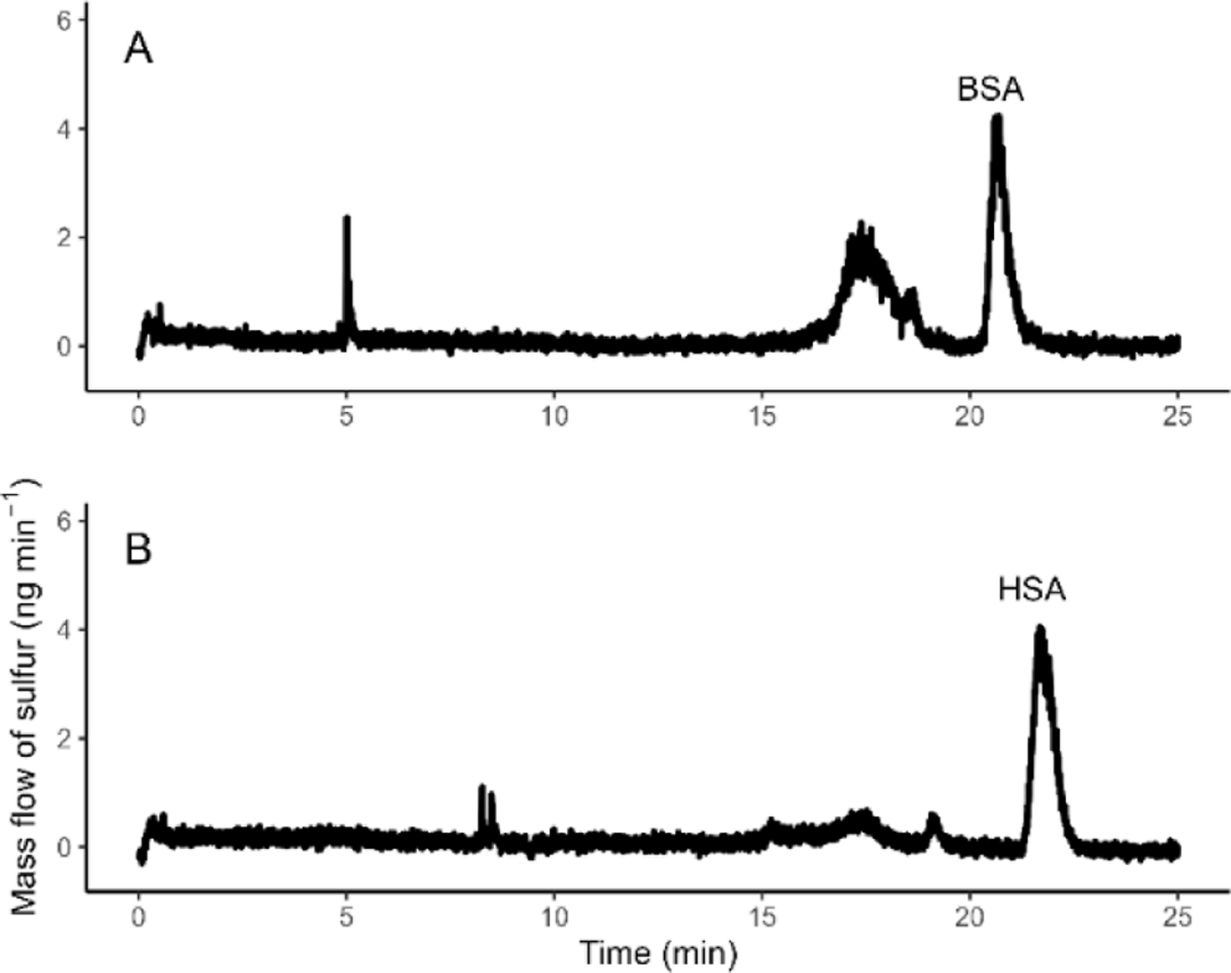
Mass flow electropherogram of serum protein separation: (A) bovine and (B) human.

To demonstrate the applicability of our online ID CE/ICP–MS method on albumin in its native matrix, we quantified BSA and HSA by diluting sera samples four times with pure water. In both samples (Figure 4), the albumin peak is baseline-separated from those of other proteins. Measured concentration of sulfur is converted to albumin concentration using the number of cysteine and methionine molecules in the AA sequence of BSA and HSA from protein knowledgebase Uniprot.org(27,28) (Table 5). The simple and reliable dyeing method with BCG was used to quantify the albumin content in sera for comparison purpose. Table 5 shows the results from both quantification approaches along with the data from the manufacturer of serum. The results agree within the uncertainty and proves applicability of our online ID CE/ICP–MS method for its purpose.

**Table 5:** Quantification Results of BSA and HSA^a^

albumin	data from Uniprot.org				measurement results		data from the manufacturer
	length of the AA sequence	albumin molar mass, Da	number of S atoms and mass fraction	ref	albumin mass concentration from online ID CE/ICP–MS, ρ ± *U*, *k* = 2 (g L^–^^1^)	albumin mass concentration from the BCG dyeing method, ρ ± *U*, *k* = 2 (g L^–^^1^)	albumin mass concentration from the electrophoretic profile (g L^–^^1^)
BSA	607	69,293	40 (35 C, 5 M), 1.85%	(27)	28.1 ± 1.8 (6.4%)	30.2 ± 3.1 (10%)	27
HSA	609	69,367	42 (35 C, 7 M), 1.94%	(28)	37.3 ± 2.6 (7.0%)	36.4 ± 3.7 (10%)	n.a.

a C—cysteine, M—methionine, and n.a.—not available.

## Conclusions

Our study successfully demonstrates that the online ID CE/ICP–MS with a species-unspecific inorganic sulfur spike is a powerful tool for the quantification of sulfur-containing biological compounds both in standard solutions and natural complex matrix. The method was optimized by using standard solutions of sulfate–albumin and cystine–methionine. Then, the method was validated using an accurately prepared mixture of albumin-depleted bovine serum spiked with pure NIST SRM 927 BSA. Finally, the applicability of the method was confirmed by the quantification of albumin in bovine (BSA) and human sera (HSA). Metrological traceability of the measured values to the SI unit was ensured by using ^34^S spike characterized by reverse ID TIMS using a primary calibrator, NIST SRM 3154, which has been measured with the NIST primary measuring system. When using the SMIL-coated capillary, the adsorption of the protein onto the CE capillary wall was significantly reduced, enabling the quantitative recovery of the compounds from the capillary and reducing the time required for washing. The LOD determined for the absolute amount of sulfur in albumin via our method (96.2 pg) was comparable with the LOD for the absolute amount of sulfur determined via another online ID method (76.9 pg), namely, via the online ID UPLC-ICP-MS/MS method for the analysis of penetratin, reported by Grønbæk-Thorsen et al.^13^ Moreover, compared to LC, with CE, the consumption of the reagents and the sample is minimal, allowing high throughput, along with reduced waste generation. Small injection volume associated with CE is highly advantageous when performing quantification analysis for clinical diagnosis using biological samples such as blood, serum, or cerebrospinal fluid from patients.

Quantification results of BSA and HSA from our method were compared to the results from the BCG dyeing method and showed an agreement within the associated measurement uncertainties. The total combined uncertainty of our online method (6.7%) was only twice higher than that of the steady-state ID ICP–MS method (3.6%). The major contributor to the uncertainty was the isotope ratio measurement (69%), which can be explained by the poor precision of calculating isotope ratios from transient signals.

When considering the application of the method with other sulfur-containing molecules, it should be noted that the preliminary identification of the molecule is necessary for an accurate conversion of the quantity. Owing to the advancements of multiple hyphenation instrumental setups,^37,38^ both qualitative and quantitative characterization of proteins can be accomplished via the combination of ICP–MS with matrix-assisted laser desorption/ionization time-of-flight MS and electrospray ionization MS. Although our study is based on a single protein model (albumin), the developed methodology can be applied to compounds with lower abundance with the integration of suitable extraction/concentration methods. Our online ID CE/ICP–MS method can be used as an accurate approach for quantification of proteins and other biological molecules via sulfur analysis in complex matrices for various fields, such as environmental, biological, and pharmaceutical studies as well as clinical diagnosis.

## References

[ref1] Stadtman E. R.; Levine R. L. Free radical-mediated oxidation of free amino acids and amino acid residues in proteins. Amino Acids 2003, 25 (3–4), 207–218. 10.1007/s00726-003-0011-2. 14661084

[ref2] Cheignon C.; Tomas M.; Bonnefont-Rousselot D.; Faller P.; Hureau C.; Collin F. Oxidative stress and the amyloid beta peptide in Alzheimer’s disease. Redox Biol. 2018, 14, 450–464. 10.1016/j.redox.2017.10.014. 29080524 PMC5680523

[ref3] Elshorbagy A. K.; Turner C.; Bastani N.; Refsum H.; Kwok T. The association of serum sulfur amino acids and related metabolites with incident diabetes: a prospective cohort study. Eur. J. Nutr. 2022, 61 (6), 3161–3173. 10.1007/s00394-022-02872-5. 35415822

[ref4] Clases D.; Ueland M.; Gonzalez de Vega R.; Doble P.; Profrock D. Quantitative speciation of volatile sulphur compounds from human cadavers by GC-ICP-MS. Talanta 2021, 221, 121424 10.1016/j.talanta.2020.121424. 33076059

[ref5] Lajin B.; Goessler W. Sulfur speciation by HPLC-ICPQQQMS in complex human biological samples: taurine and sulfate in human serum and urine. Anal. Bioanal. Chem. 2018, 410 (26), 6787–6793. 10.1007/s00216-018-1251-z. 30062511 PMC6132542

[ref6] Suzuki Y.; Nobusawa A.; Furuta N. Quantification of proteins by measuring the sulfur content of their constituent peptides by means of nano HPLC-ICPMS. Anal. Sci. 2014, 30 (5), 551–559. 10.2116/analsci.30.551. 24813953

[ref7] Rampler E.; Dalik T.; Stingeder G.; Hann S.; Koellensperger G. Sulfur containing amino acids - challenge of accurate quantification. J. Anal. At. Spectrom. 2012, 27 (6), 1018–1023. 10.1039/c2ja10377j.

[ref8] Leonhard P.; Prange A.; Prange A. Determination of sulfur and selected trace elements in metallothionein-like proteins using capillary electrophoresis hyphenated to inductively coupled plasma mass spectrometry with an octopole reaction cell. Anal. Bioanal. Chem. 2003, 377 (1), 132–139. 10.1007/s00216-003-2041-8. 12898119

[ref9] Yeh C.-F.; Jiang S.-J.; Hsi T.-S. Determination of sulfur-containing amino acids by capillary electrophoresis dynamic reaction cell inductively coupled plasma mass spectrometry. Anal. Chim. Acta 2004, 502 (1), 57–63. 10.1016/j.aca.2003.09.051.

[ref10] Feng L.; Huo Z.; Xiong J.; Li H. Certification of Amyloid-Beta (Aβ) Certified Reference Materials by Amino Acid-Based Isotope Dilution High-Performance Liquid Chromatography Mass Spectrometry and Sulfur-Based High-Performance Liquid Chromatography Isotope Dilution Inductively Coupled Plasma Mass Spectrometry. Anal. Chem. 2020, 92 (19), 13229–13237. 10.1021/acs.analchem.0c02381. 32847351

[ref11] Wang M.; Feng W.; Lu W.; Li B.; Wang B.; Zhu M.; Wang Y.; Yuan H.; Zhao Y.; Chai Z. Quantitative analysis of proteins via sulfur determination by HPLC coupled to isotope dilution ICPMS with a hexapole collision cell. Anal. Chem. 2007, 79 (23), 9128–9134. 10.1021/ac071483t. 17958377

[ref12] Giner Martínez-Sierra J.; Moreno Sanz F.; Herrero Espílez P.; Santamaria-Fernandez R.; Marchante Gayón J. M.; García Alonso J. I. Evaluation of different analytical strategies for the quantification of sulfur-containing biomolecules by HPLC-ICP-MS: Application to the characterisation of 34S-labelled yeast. J. Anal. At. Spectrom. 2010, 25 (7), 989 10.1039/b925366a.

[ref13] Grønbæk-Thorsen F.; Stürup S.; Gammelgaard B.; Møller L. H. Development of a UPLC-IDA-ICP-MS/MS method for peptide quantitation in plasma by Se-labelling, and comparison to S-detection of the native peptide. J. Anal. At. Spectrom. 2019, 34 (2), 375–383. 10.1039/C8JA00341F.

[ref14] Schaier M.; Hermann G.; Koellensperger G.; Theiner S. Accurate characterization of β-amyloid (Aβ40, Aβ42) standards using species-specific isotope dilution by means of HPLC-ICP-MS/MS. Anal. Bioanal. Chem. 2022, 414 (1), 639–648. 10.1007/s00216-021-03571-6. 34355254 PMC8748378

[ref15] Schaumlöffel D.; Prange A.; Marx G.; Heumann K. G.; Bratter P. Characterization and quantification of metallothionein isoforms by capillary electrophoresis-inductively coupled plasma-isotope-dilution mass spectrometry. Anal. Bioanal. Chem. 2002, 372 (1), 155–163. 10.1007/s00216-001-1164-z. 11939186

[ref16] Pritzkow W.; Vogl J.; Köppen R.; Ostermann M. Determination of sulfur isotope abundance ratios for SI-traceable low sulfur concentration measurements in fossil fuels by ID-TIMS. Int. J. Mass Spectrom. 2005, 242 (2–3), 309–318. 10.1016/j.ijms.2004.10.024.

[ref17] Faßbender S.; Rodiouchkina K.; Vanhaecke F.; Meermann B. Method development for on-line species-specific sulfur isotopic analysis by means of capillary electrophoresis/multicollector ICP-mass spectrometry. Anal. Bioanal. Chem. 2020, 412 (23), 5637–5646. 10.1007/s00216-020-02781-8. 32613566 PMC8236454

[ref18] Seam N.; Gonzales D. A.; Kern S. J.; Hortin G. L.; Hoehn G. T.; Suffredini A. F. Quality control of serum albumin depletion for proteomic analysis. Clin. Chem. 2007, 53 (11), 1915–1920. 10.1373/clinchem.2007.091736. 17890439

[ref19] McPherson I. G.; Everard D. W. Serum albumin estimation: Modification of the bromocresol green method. Clin. Chim. Acta 1972, 37, 117–121. 10.1016/0009-8981(72)90422-6. 5022074

[ref20] Walsh R. L. A comparison of dye binding methods for albumin determination: the effects of abnormal sera, reaction times, acute phase reactants and albumin standards. Clin. Biochem. 1983, 16 (3), 178–181. 10.1016/S0009-9120(83)90231-X. 6851081

[ref21] Rodriguez-Gonzalez P.; Epov V. N.; Pecheyran C.; Amouroux D.; Donard O. F. Species-specific stable isotope analysis by the hyphenation of chromatographic techniques with MC-ICPMS. Mass Spectrom. Rev. 2012, 31 (4), 504–521. 10.1002/mas.20352. 22161869

[ref22] Tukhmetova D.; Lisec J.; Vogl J.; Meermann B. Data processing made easy: standalone tool for automated calculation of isotope ratio from transient signals - IsoCor. J. Anal. At. Spectrom. 2022, 37 (11), 2401–2409. 10.1039/D2JA00208F.

[ref23] Rottmann L.; Heumann K. G. Development of an on-line isotope dilution technique with HPLC/ICP-MS for the accurate determination of elemental species. Fresenius’ J. Anal. Chem. 1994, 350 (4–5), 221–227. 10.1007/bf00322473.

[ref24] Vogl J.; Pritzkow W. Isotope dilution mass spectrometry — A primary method of measurement and its role for RM certification. Mapan 2010, 25 (3), 135–164. 10.1007/s12647-010-0017-7.

[ref25] Irrgeher J.; Vogl J.; Santner J.; Prohaska T. Measurement Strategies. In Sector Field Mass Spectrometry for Elemental and Isotopic Analysis; Prohaska T., Irrgeher J., Eds.; The Royal Society of Chemistry: Cambridge, 2015, pp 126–151.

[ref26] CIAAW Natural variations of isotopic abundances 2007–2015. https://ciaaw.org/natural-variations.htm (accessed April 28, 2023).

[ref27] UniProt: the Universal Protein Knowledgebase in 2023: P02769 · ALBU_BOVIN. https://www.uniprot.org/uniprotkb/P02769/entry (accessed April 29 2023).

[ref28] UniProt: the Universal Protein Knowledgebase in 2023: P02768 · ALBU_HUMAN. https://www.uniprot.org/uniprotkb/P02768/entry (accessed April 29 2023).

[ref29] Evaluation of Measurement Data—Guide to the Expression of Uncertainty in Measurement JCGM 100:2008: 2008.

[ref30] Lemke N.; El-Khatib A. H.; Tchipilov T.; Jakubowski N.; Weller M. G.; Vogl J. Procedure providing SI-traceable results for the calibration of protein standards by sulfur determination and its application on tau. Anal. Bioanal. Chem. 2022, 414 (15), 4441–4455. 10.1007/s00216-022-03974-z. 35316347 PMC9142460

[ref31] Keren D. F. Capillary zone electrophoresis in the evaluation of serum protein abnormalities. Am. J. Clin. Pathol. 1998, 110 (2), 248–252. 10.1093/ajcp/110.2.248. 9704625

[ref32] Petersen J. R.; Okorodudu A. O.; Mohammad A.; Payne D. A. Capillary electrophoresis and its application in the clinical laboratory. Clin. Chim. Acta 2003, 330 (1–2), 1–30. 10.1016/S0009-8981(03)00006-8. 12636924

[ref33] Bossuyt X.; Lissoir B.; Marien G.; Maisin D.; Vunckx J.; Blanckaert N.; Wallemacq P. Automated Serum Protein Electrophoresis by Capillarys. Clin. Chem. Lab. Med. 2003, 41 (5), 704–710. 10.1515/cclm.2003.107. 12812271

[ref34] Leclercq L.; Renard C.; Martin M.; Cottet H. Quantification of Adsorption and Optimization of Separation of Proteins in Capillary Electrophoresis. Anal. Chem. 2020, 92 (15), 10743–10750. 10.1021/acs.analchem.0c02012. 32598142

[ref35] Luo D.; Smith S. W.; Anderson B. D. Kinetics and mechanism of the reaction of cysteine and hydrogen peroxide in aqueous solution. J. Pharm. Sci. 2005, 94 (2), 304–316. 10.1002/jps.20253. 15570599

[ref36] Mishra V.; Heath R. J. Structural and Biochemical Features of Human Serum Albumin Essential for Eukaryotic Cell Culture. Int. J. Mol. Sci. 2021, 22 (16), 8411 10.3390/ijms22168411. 34445120 PMC8395139

[ref37] Coufalikova K.; Benesova I.; Vaculovic T.; Kanicky V.; Preisler J. LC coupled to ESI, MALDI and ICP MS - A multiple hyphenation for metalloproteomic studies. Anal. Chim. Acta 2017, 968, 58–65. 10.1016/j.aca.2017.03.016. 28395775

[ref38] Pröfrock D. Hyphenation of capillary-LC with ICP-MS and parallel on-line micro fraction collection for MALDI-TOF-TOF analysis—complementary tools for protein phosphorylation analysis. J. Anal. At. Spectrom. 2010, 25 (3), 334 10.1039/b921145d.

